# Familial risk and diagnosis of colorectal cancer in young individuals

**DOI:** 10.1186/1897-4287-13-S1-A6

**Published:** 2015-09-09

**Authors:** Pavel Elsakov

**Affiliations:** 1 State Research Institute, Innovative Medicine Center, Vilnius, Lithuania

## 

The age standardized (world) incidence (per 100000) of large bowel cancer in Lithuania is increasing: in 1993-1997 it was 25.6 for males and 16.8 for females; in 1998-2002 it was 30.4 and 19.1 respectively. Meanwhile the age standardized (world) cancer mortality remains without change: in the same periods it was 18.0 for males and 11.5 for females; 18.6 and 10.6 respectively. These epidemiological aspects are basically for start CRC screening in national population. The age for population screening recommended by EC is 50-74 years and the optimal participation rate to get better survival of CRC patients is 45-60% of population. Numerous studies have shown the risk of getting CRC is higher if a first-degree family member had the disease, and shows that your chances of surviving the disease may be influenced in part by your family ties. It hypothesized that patients with a family history of CRC might be more likely to get screened for the disease, finding tumors earlier, and are more likely to have a better prognosis. In addition to collecting family tree information help for recognition of hereditary cancer syndrome such as HNPCC, Peutz-Jeghers Polyposis, and FAP. The familial risk for CRC and effective cancer prevention become more important in individuals aged below 50-55 years. The study in Lithuania was aimed to prove more knowledge on familial CRC risk and the surveillance of family members at risk for large bowel malignancy. This study indicates that a positive family history of CRC can be recognized as a prognostic factor for individuals aged 25-39 years. Results shows that a positive family history was not a clear indicator of early stage diagnosis in the groups studied compared to the population.

